# Mandibular sporadic Burkitt lymphoma in an adult patient: A case report and review of the literature

**DOI:** 10.1002/ccr3.4535

**Published:** 2021-07-21

**Authors:** Nahal Azimi, Farnoosh Razmara, Samira Derakhshan, Neda Kardouni Khoozestani

**Affiliations:** ^1^ School of Dentistry International Campus Tehran University of Medical Sciences Tehran Iran; ^2^ Department of Oral and Maxillofacial Surgery School of Dentistry Tehran University of Medical Sciences Tehran Iran; ^3^ Craniomaxillofacial Research Center Tehran University of Medical Sciences Tehran Iran; ^4^ Department of Oral and Maxillofacial Pathology School of Dentistry Tehran University of Medical Sciences Tehran Iran; ^5^ Cancer Institute Tehran University of Medical Sciences Tehran Iran

**Keywords:** Burkitt lymphoma, head and neck neoplasms, lymphoma, mandible, non‐Hodgkin, oral lymphoma, sporadic

## Abstract

Only a minor percent of lymphomas arise in the oral cavity. Although rare, dentists and clinicians should not neglect them as a possible consideration in the differential diagnosis of oral lesions.

## Graphical abstract

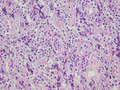

## 1 INTRODUCTION

Non‐Hodgkin's lymphoma (NHL) is a malignant neoplasm of the lymphoid tissue and is one of the most prevalent cancers seen worldwide.^1^ A rare and aggressive variant of this neoplasm, Burkitt lymphoma (BL), develops from germinal or post‐germinal B cells and is characterized by *MYC* oncogene translocation.^2^

BL is further classified into 3 epidemiologically dissimilar types with different population targets: endemic‐, sporadic‐, and immunodeficiency‐associated BL.^3^ The sporadic subtype which yearly has an incidence of around 3 cases out of a million people, typically occurs in children and comprises less than 1% of all adult NHLs.^4^

Only 8.9% of the head and neck neoplasms are lymphomas, 1.9% of them occurring in oral cavity.^5^ Oral lymphomas usually occur in the tonsils, palate, gingiva, or jaws and may be seen centrally in bone or peripherally in soft tissue. Most of them are “diffuse large B‐cell lymphomas” (DLBCL) or plasmablastic lymphomas, the latter being frequent in immunodeficient patients.^6^, ^7^ Endemic BL also involves the head and neck site especially the jaws frequently. However, sporadic BL rarely has a presentation in this region.^8^ In this case report, we present an adult patient with sporadic BL involving the mandible.

## 2 CASE REPORT

**FIGURE 1:**
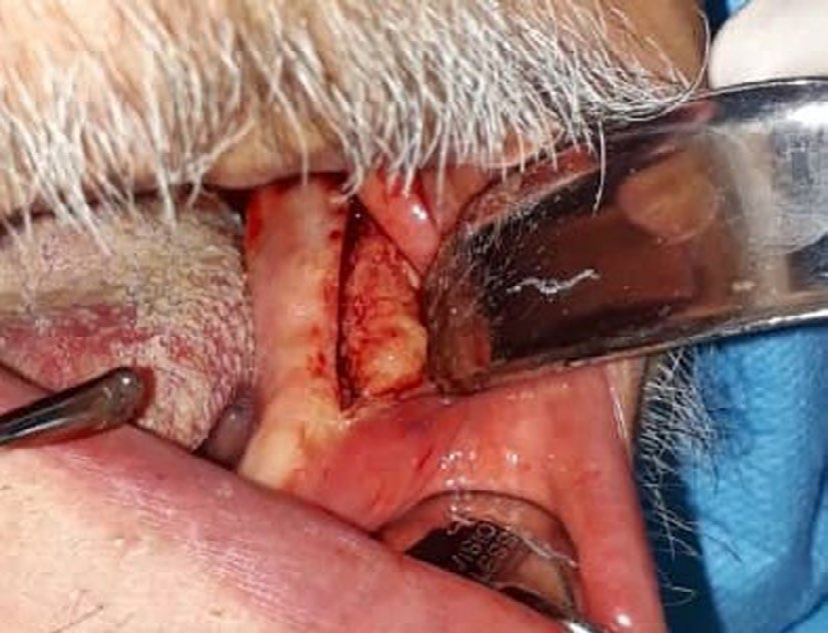
The clinical photograph reveals a distinct swelling on the left side of the mandible

A 49‐year‐old white man was referred to the maxillofacial surgery department of Tehran University in July 2019 with a chief complaint of vague pain in the left mandibular area and lip paresthesia. He reported occasional episodes of fever, otherwise, there was no further significant finding in his medical history. On clinical examination, a mass with intact overlying mucosa and firm consistency was found at the left edentulous mandibular ridge (Figure 1).

**FIGURE 2:**
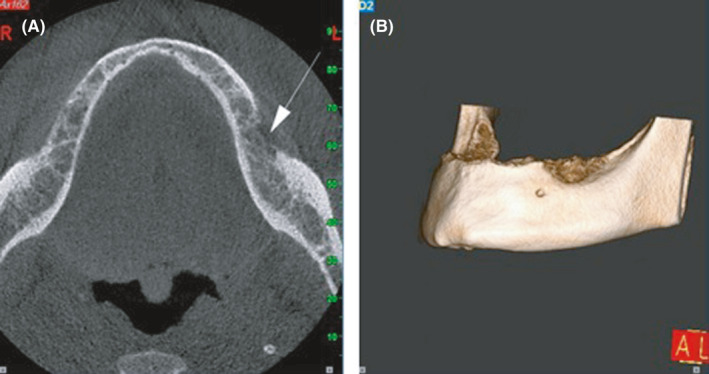
(A) Axial view of cone beam computed tomography (CBCT) shows an ill‐defined mass with buccal cortical plate invasion. (B) Three‐dimensional CBCT demonstrates left‐sided osteolytic destruction of the mandible

Based on the clinical features of the lesion, that is, firm consistency and intact surface mucosa, a spectrum of differential diagnosis from a benign long‐standing mesenchymal lesion such as ancient schwannoma to a borderline or low‐grade mesenchymal malignancy such as fibrosarcoma was proposed. Cone beam computed tomography (CBCT) was performed which revealed a left‐sided, ill‐defined radiolucent lesion extending from the left alveolar crest to the inferior alveolar canal of the mandible with left buccal cortical plate invasion (Figure 2).

**FIGURE 3:**
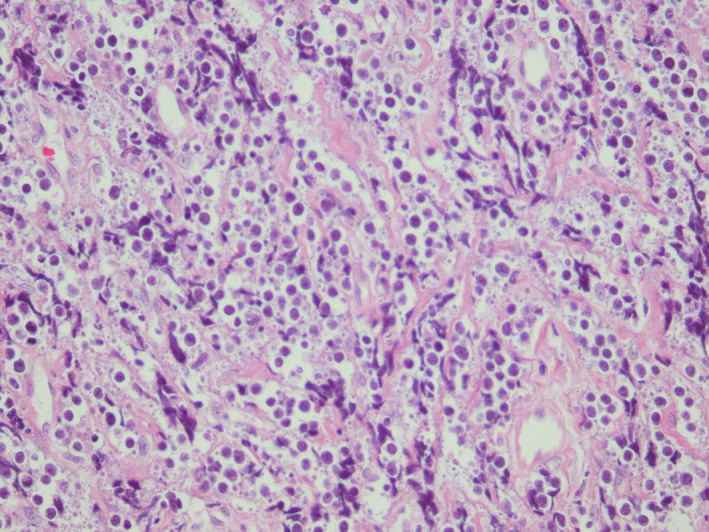
H&E section of the left mandibular ridge incisional biopsy. Small to medium‐sized malignant lymphocytes with round nuclei are observed in a monotonous pattern (×400 magnification)

**FIGURE 4:**
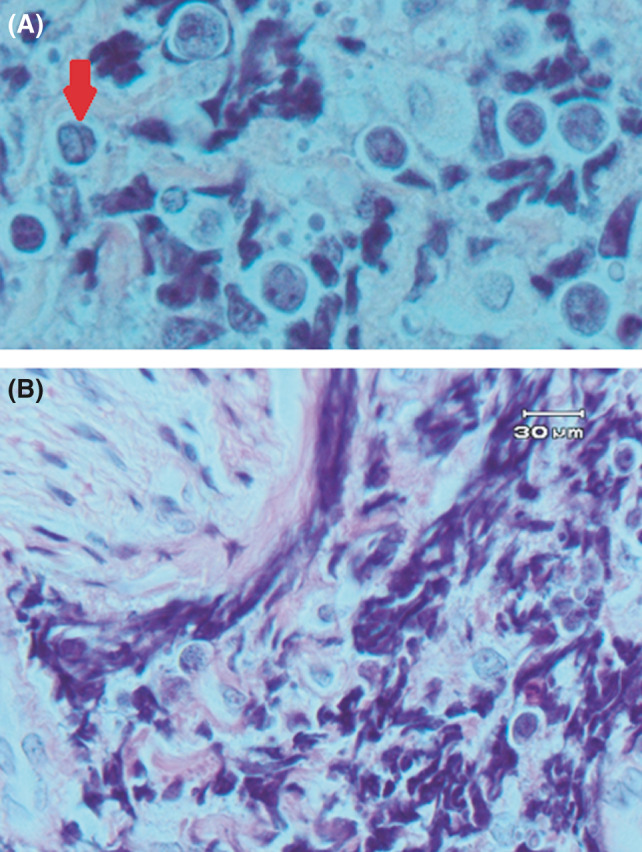
H&E sections. (A) Tumoral cells with clefted nuclei (×1000 magnification). (B) Perineural invasion of tumoral cells (×1000 magnification)

**FIGURE 5:**
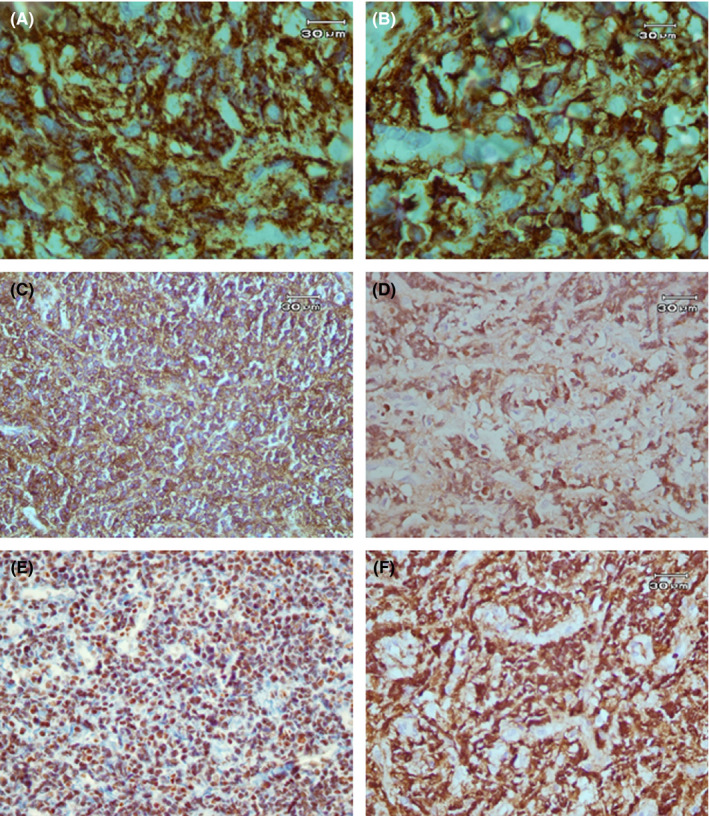
Immunohistochemistry sections. (A) CD20 positive staining of B cells (×1000 magnification). (B) CD45 positive immunostaining (×1000 magnification). (C) CD10 positive immunoreactivity (×400 magnification). (D) Nuclear BCL‐6 staining (×400 magnification). (E) C‐MYC positive immunostaining (×400 magnification). (F) The majority of cells show a Ki67 proliferation index positivity (×400 magnification)

An incisional biopsy was carried out under local anesthesia. The gross specimen consisted of four pieces of tan irregular tissue with soft to elastic consistency measuring 3.4 × 1.6 × 0.5 centimeters totally. Microscopic examination of the lesion demonstrated a malignant round cell tumor composed of hyperchromatic, small to medium‐sized round cells with round and occasionally clefted nuclei, prominent nucleoli, and scant cytoplasm forming large sheets (Figures 3 and 4A). Easily identifiable mitotic figures, perineural invasion, and crush artifacts were also observed (Figure 4B). Immunohistochemistry (IHC) findings of the *paraffin*‐*embedded* tissue revealed a positive reaction to immunostains CD45, CD20, C‐MYC, CD10, and BCL‐6, a scattered interstitial positive reactivity to CD3 while, no expression was detected for CD5, BCL‐2, Cyclin D1, CD19, terminal nucleotidyltransferase (TdT), and myeloperoxidase (MPO) (Figure 5). The proliferative activity based on Ki67 immunostaining was more than 95% and the *In Situ* Hybridization for Epstein–Barr virus (EBV) tested negative (Figure 5).

The patient was referred to the oncology and hematology department. A complete blood count was conducted demonstrating normocytic normochromic anemia. Upon physical examination, hepatosplenomegaly was detected. On whole‐body Tc99m‐MDP bone scan which was performed simultaneously, areas of focally increased radiotracer uptake on both sides of the mandible were detected. No evidence of metabolically active bony lesion or active inflammatory process was seen in other regions of the skeletal system. A computed tomography scan of the chest and abdomen revealed no involvement.

To elucidate bone marrow involvement, bone marrow biopsy and touch preps were performed. The marrow cellularity was more than 80% with scattered isolated preserved fat cells. The marrow was considerably infiltrated by monomorphous lymphoid cells with a diffuse growth pattern. The infiltration was constituted of small to medium‐sized lymphoid cells with irregular nuclei. A focal "starry‐sky" effect was also observed. Preserved hematopoietic elements were mostly absent. The touch prep also showed small to medium‐sized lymphoid cells with rounded and scattered clefted nuclei, small nucleoli, and scanty cytoplasm.

Overall, findings were congruent with the diagnosis of BL of the mandible. The patient was categorized as stage IVB and an intensive chemotherapeutic regimen was prescribed for him. Bone marrow aspiration and trephine biopsy, performed 3 months after the initial diagnosis, revealed hypercellular marrow with mild eosinophilia (12%) and no marrow involvement.

## 3 LITERATURE REVIEW

We searched PubMed and Scopus databases to review cases of intraoral sporadic BL in adult immunocompetent patients. Articles available in full‐text since 2000 were reviewed. Patient data including age, gender, clinical presentation and complaints, tumor location, radiographic features, EBV detection, abdomen involvement, treatment, and outcome are summarized in Table 1.

**TABLE 1:** Sporadic Burkitt lymphoma of the oral cavity in adult immunocompetent patients’ literature review

First Author‐year	Age‐Sex	Clinical Presentation and Presenting Complaint	Location	Panoramic or Peri‐Apical Radiographic Features	EBV	Abdominal involvement	Therapy	Outcome	Follow‐up Duration
Parker^26^ 2020	37‐F	Painful swelling +perioral paresthesia	Mandible	Well‐defined uni‐locular radioluceny.	NFS	Yes	ChT	DF	NFS
Tseng^11^ 2020	84‐M	Swelling	Mandibular gingiva	Osteolysis	NFS	No	Hospital care	DOD	1 mo
Pedraza^27^ 2019	63‐M	Bilateral, aching erythematous mass	Mandible	NFS	+	Yes	Patient died before therapy	DOD	10 days
Garcia^28^ 2017	42‐M	Mild swelling +lip paresthesia +tooth mobility	Mandible	No changes	NFS	NFS	ChT	DF	4 yr
Kuo^29^ 2017	29‐M	2 ulcerated and discrete swellings	Mandibular gingiva +retromolar pad	One Lesion showed well‐defined uni‐locular radiolucency.	+	NFS	ChT +RT	DF	5 yr
Goto^30^ 2016	27‐F	Painful area +lip paresthesia	Mandible	Osteolysis	NFS	NFS	ChT	DF	NFS
Patankar^31^ 2015	38‐M	Painful swelling +tooth mobility and displacement	Mandibular and particularly maxillary gingiva	No changes	NFS	NFS	Patient died before therapy	DOD	Few days
Rebelo‐Pontes^32^ 2014	54‐F	Swelling +Tooth mobility, separately	Mandible	Root resorption +lamina dura loss +osteolysis	+	Yes	ChT	DF	2 yr
Rebelo‐Pontes^32^ 2014	22‐M	Painful exophytic mass	Mandible	Root resorption +lamina dura loss +osteolysis	+	Yes	ChT	DF	1 yr
Barboza^33^ 2013	35‐M	Painless ulcerated mass	Maxilla	ill‐defined radiolucency	‐	No	ChT	Considerable remission	6 mo
Boffano^34^ 2013	35‐M	Bilateral painless and ulcerated swelling	Maxilla	NA	‐	Yes	ChT	NFS	NFS
Kikuchi^35^ 2012	61‐F	Lip paresthesia +painful area	Mandible	No changes	‐	No	ChT	DF	11 mo
Balasubrama niam^36^ 2009	36‐F	Painful, bleeding, ulcerated swelling	Mandible	PDL widening +slight ill‐defined alveolar bone loss	‐	No	ChT	DF	3 mo
Cascarini^37^ 2005	38‐M	Painful swelling +lip paresthesia	Mandible	No changes	NFS	No	ChT	DF	3 yr
Landesberg^14^ 2001	28‐M	Lip paresthesia +painful area	Mandible and Maxilla	No changes	‐	Yes	ChT	DF	16 mo
Yoskovitch^38^ 2000	76‐M	Submucosal mass causing dysphagia and odynophagia	Dorsal surface of the tongue	NA	NFS	No	ChT	DF^a^	18 mo
Present Case 2021	49‐M	Painful mass +lip paresthesia	Mandible	ill‐defined uni‐locular radiolucency	‐	No	ChT	DF	3 mo

Abbreviations: ChT, Chemotherapy; DF, Disease‐free; DOD, Died of the disease; mo, month; NA, not applicable; NFS, not further specified; RT, Radiation therapy; yr, year.

^a^ Despite an initial regression following ChT, lesion increased in size after a few months. Therefore, a second course of ChT was performed leading to complete remission of this patient.

The average age of the 17 adult cases was 44.3 years ranging from 22 to 84 with the median age of 38. Nearly, 70% of the patients were men and 30% were female. Among patients with a single jaw involved, 12 had mandibular lesions while maxillary involvement was observed in only 2 cases. Out of 14 patients receiving cancer treatment, chemotherapy as a sole treatment was performed for 13 whereas, only one underwent a chemoradiotherapy regimen. Eventually, 80% became disease‐free while 20% died of the disease (out of 15 cases with a known outcome).

## 4 DISCUSSION

Based on WHO classification, 3 variants have been defined for BL: endemic‐, sporadic‐ or non‐endemic‐, and the immunodeficiency‐associated type. Being the most prevalent variant, endemic BL is mostly seen in equatorial Africa. This form predominantly affects children between 4 and 7 years old with a male preponderance.^9^

The immunodeficiency‐associated variant affects patients with HIV or organ transplantation.^2^ Patients in this category have a more progressive disease and are also more likely to develop B symptoms or bone marrow involvement.^2^

Sporadic BL constitutes less than 3% of all NHL cases, being more prevalent among youngsters. This variant may occur in any world region but is typically observed in Europe and North America.^3^, ^9^ It demonstrates a male proclivity, which is consistent with the demographic features of our case and literature review.^9^

The facial skeleton particularly the jaws are the most commonly involved sites in endemic BL. However, in non‐endemic variant, the main site of involvement is the abdomen. Mandibular involvement in the latter variant as observed in our case is a rare finding and maxillary involvement is even less seen.^9^, ^10^

Mandible bone swelling is the most common clinical feature of mandibular lymphomas including BL presenting as a painful area with tooth mobility.^10^, ^11^ Mobility and pain history may lead the clinician toward the misdiagnosis of odontogenic infections causing patients to undergo unnecessary dental treatments.^12^ However, this was not a matter of concern for our patient since he was completely edentulous. Another common clinical symptom is mental nerve neuropathy or “numb chin syndrome” complained as chin and lower lip paresthesia.^10^, ^13^ Apart from malignancies, this symptom may be seen following anesthetic injection, trauma, or severe atrophy of the mandibular ridge, and is also observed in patients with multiple sclerosis and diabetes mellitus.^10^

Bone involvement while no clear odontogenic infection is detected, is also considered as a crucial element of jaw tumors. Non‐specific osteolytic lesions with ill‐defined borders are the most common radiographic presentation of oral lymphomas followed by PDL widening, tooth displacement, and lamina dura loss. However, some BL intraosseous lesions have been reported with no radiographic alterations. This may be due to the fact that the tumor is confined to the medullary bone.^14^ In our case, the extension of bone destruction on the left side from the crest of the mandible to the inferior alveolar canal may be an indication of the patient's symptoms.

Microscopic features of our case were compatible with malignant round cell tumors including NHL so, we decided to perform an IHC panel. Since there were positive reactions to CD45 and CD20 and a weak immunoreaction to CD3, an NHL of B‐cell lineage was determined. TdT negativity ruled out B‐cell lymphoblastic lymphoma and, a positive reaction to CD10 together with negative reactivity to CD5, and BCL‐2 were not in favor of B‐cell “chronic lymphocytic leukemia/ small lymphocytic lymphoma”. Plus, CD5 and Cyclin D1 negativity precluded a mantle cell lymphoma diagnosis. A positive reaction to germinal B‐cell biomarkers, CD10 and BCL‐6, guided us to lymphomas originating from the germinal center: DLBCL, BL, and follicular lymphoma.^15^

Histomorphologic features in our case such as the homogenous pattern of small to medium‐sized cells, lack of appreciable large cells, absence of any considerable nuclear polymorphism, "starry‐sky" pattern, and the very high (>95%) Ki67 index were compatible with BL. Moreover, CD10 and BCL‐6 positivity and BCL‐2 negativity which are the typical immunophenotype of BL, were in line with this diagnosis.^16^

The other differential diagnosis, in this case, was "B‐cell lymphoma, unclassifiable, with features intermediate between DLBCL and classical BL". This category has been called "high‐grade B‐cell lymphoma with rearrangements of *MYC* and *BCL*‐*2* and/or *BCL*‐*6*" in the 2016 WHO classification of lymphomas, being consisted of mostly double‐hit and triple‐hit lymphomas (DHL, THL). Translocation of *MYC*, as well as *BCL*‐*2* or *BCL*‐*6*, would be named DHL while rearrangement in all three is identified as THL.^17^ In these cases, the tumor may be histomorphologically compatible with BL while demonstrating atypical immunophenotypic features such as a positive reaction to *BCL*‐*2* or a relatively lower Ki67 expression, or the tumor may have the phenotypic characteristics of BL with an abnormal histomorphology like the presence of large‐sized cell populations.^18^ However, none of these two situations were evident in the presented case.

It should be noted that before making DHL or THL as a definite diagnosis, the patient should be screened for *MYC*, *BCL*‐*2*, and *BCL*‐*6* translocations by fluorescent *in situ* hybridization (FISH) test. However, due to the unavailability of this test in some laboratories, a possible approach is to initially perform IHC on CD10 and BCL‐2 markers. If both show a positive result, then FISH must be carried out on *MYC* oncogene. Assessment of *BCL*‐*2* and *BCL*‐*6* rearrangements by FISH is warranted for cases demonstrating *MYC* translocation.^17^

Naresh et al also provided a scoring algorithm for distinguishing BL, DLBCL, and cases with intermediate features, by considering the morphological characteristics and a panel of IHC markers. In the first phase of this algorithm, in which only morphology, BCL‐2, and CD10 expression are evaluated, our case obtained a sufficient score for the diagnosis of BL. However, in more complicated cases, the clinician steps further in the algorithm requiring the assessment of more IHC markers or FISH test to reach an accurate diagnosis.^19^

Another differential diagnosis, in this case, was diffuse follicular lymphoma. However, weighing up the IHC results and considering the following facts, this subtype was ruled out: (1) Follicular lymphoma accounts for only 6.1% of extranodal NHLs.^20^ (2) An extremely high‐Ki‐67 proliferation index is less likely in this subtype.^21^ (3) There was no follicular pattern at all in our case.^21^ (4) BCL‐2 is positive in 50%–70% of high‐grade follicular lymphomas and also a reduction in CD10 expression may be observed.^22^

While almost all patients with endemic BL have EBV infection, this virus has been found in only 25–40% of the sporadic subtype of BL patients.^8^ Satou et al stated that the EBV‐positive sporadic form has a greater age distribution compared to the EBV‐negative sporadic variant and there is no remarkable difference in the overall survival rate between the two groups.^23^

Since this tumor has an aggressive behavior, immediate histopathologic and cytogenetic evaluation and early treatment are essential.^24^ Due to the paucity of the literature and clinical trials on BL in adults, there is a controversy over the most appropriate treatment for these patients.^3^, ^8^ Generally, this tumor is highly sensitive to chemotherapy therefore, short, dose‐intensive systemic chemotherapy regimens are warranted.^25^

To sum up, rapid osseous destruction with no odontogenic origin, or unexplained chronic pain and paresthesia in the mandible may be early signs of a more serious condition, possibly a neoplasm of which dentists should be aware and take into consideration.

## CONFLICT OF INTEREST

None declared.

## AUTHOR CONTRIBUTIONS

NA: Performed data collection and wrote the manuscript. FR: Was involved in the conception and design of the study, performed the surgical procedure, and revised the manuscript. SD: Evaluated the histopathologic results, supervised the laboratory steps, and reviewed the manuscript. NKK: Was involved in the conception and design of the study, evaluated the histopathologic results, supervised the laboratory steps and revised the manuscript.

## ETHICAL STATEMENT

Published with the patient's written consent.

## Data Availability

The data that support the findings of this study are available from the corresponding author upon reasonable request.
